# Therapeutic Potential of Quercetin as an Antioxidant for Bone-Muscle-Tendon Regeneration and Aging

**DOI:** 10.14336/AD.2024.0282

**Published:** 2025-06-24

**Authors:** Jae Gyu Kim, Ashish Ranjan Sharma, Yeon-Hee Lee, Srijan Chatterjee, Yean Jung Choi, Roshani Rajvansh, Chiranjib Chakraborty, Sang-Soo Lee

**Affiliations:** ^1^Institute for Skeletal Aging & Orthopedic Surgery, Hallym University-Chuncheon Sacred Heart Hospital, Chuncheon-si, 24252, Gangwon-do, Korea.; ^2^Department of Food and Nutrition, Sahmyook University, Seoul 01795, Korea.; ^3^Department of Biotechnology, School of Life Science and Biotechnology, Adamas University, Barasat-Barrackpore Road, Kolkata, West Bengal 700126, India.

**Keywords:** Quercetin, Bioflavonoid, Oxidative stress, Musculoskeletal aging

## Abstract

Quercetin (QC), a naturally occurring bioflavonoid found in various fruits and vegetables, possesses many potential health benefits, primarily attributed to its robust antioxidant properties. The generation of oxidative stress in bone cells is a key modulator of their physiological behavior. Moreover, oxidative stress status influences the pathophysiology of mineralized tissues. Increasing scientific evidence demonstrates that manipulating the redox balance in bone cells might be an effective technique for developing bone disease therapies. The QC antioxidant abilities in skeletal muscle significantly enhance muscle regeneration and reduce muscle atrophy. In addition, QC has been shown to have protective effects against oxidative stress, inflammation, apoptosis, and matrix degradation in tendons, helping to maintain the structural integrity and functionality of tendons. Thus, the antioxidant properties of QC might be crucial for addressing age-related musculoskeletal disorders like osteoporosis, sarcopenia, and tendon-related inflammatory conditions. Understanding how QC influences redox signaling pathways involved in musculoskeletal disorders, including their effect on bone, muscle, and tendon differentiation, might provide insights into the diverse advantages of QC in promoting tissue regeneration and preventing cellular damage. Therefore, this study reviewed the intricate relationship among oxidative stress, inflammation, and tissue repair, affected by the antioxidative abilities of QC, in age-related musculoskeletal tissues to improve the overall health of bones, muscles, and tendons of the skeletal system. Also, reviewing the ongoing clinical trials of QC for musculoskeletal systems is encouraging. Given the positive effect of QC on musculoskeletal health, further scientific investigations and controlled human intervention studies are necessary to explore the therapeutic potential to its optimum strength.

## 1. Introduction

The musculoskeletal system, comprised of skeletal muscle and bone tissue, is crucial for facilitating voluntary movement. A probable coexistence of sarcopenia and osteoporosis is indicated by the fact that decreased muscle mass and strength are frequently associated with decreases in bone mass. Exposure to such conditions can result in a decline in patients' quality of life and a higher mortality risk. Lately, skeletal muscle has been recognized as an endocrine organ capable of affecting other tissues [1]. In addition, it is worth noting that pelvic and sacral fractures caused by low-impact trauma in older adults have unique characteristics that set them apart from the pelvic ring injuries commonly seen in younger individuals [2]. The tendon is a vital component of the musculoskeletal system. They are crucial in linking muscles and bones, enabling the efficient transfer of forces necessary for bodily movement. Tendon injuries, whether acute or chronic, are relatively common among people and can cause significant pain and functional impairment. Managing tendon injuries can be reasonably challenging for clinicians [3]. Ensuring the balance and stability of muscular function is essential for survival and well-being. Skeletal muscle wasting not only dramatically reduces the standard of life and enhances illness and death rates but also places a substantial economic burden. The search for a viable therapy for skeletal muscle wastage is challenging because our knowledge of its molecular causes is still insufficient [4]. Osteoporosis is a persistent bone condition defined by irregular bone structure and reduced bone density, resulting in diminished bone mass and an increased susceptibility to brittle fractures. Bisphosphonates, namely anti-resorptive medicines, are presently the principal form of treatment in numerous developing nations. Nevertheless, these treatments have restrictions and negative consequences, which have led to the creation of anabolic medications like teriparatide and romosozumab [5]. Tendinopathy refers to painful conditions in and around tendons due to overuse, presenting complex and multifactorial pathologies. Despite recent advancements, treating tendinopathy remains challenging as its definitions, risk factors, and pathophysiology continue to evolve [6]. Therefore, the limitations in treating these diseases underscore the need for new therapeutic agents.

Flavonoids are usually found in fruits, like apples, and vegetables, such as onions. These compounds are commonly used in nutritional supplements and are essential for maintaining human health and well-being. The compounds are widely recognized for their diverse range of bioactivities, including the ability to act as an antioxidant, anti-allergic, anti-inflammatory, induce anti-cancer effects, stimulate bone metabolism, and combat viral infections [7, 8]. There has been a significant surge of interest in the scientific community regarding the role of flavonoids in bone remodeling. Many flavonoids have been found to affect crucial elements in the primary bone signaling pathways, like WNT and bone morphogenetic protein (BMP) signaling [9, 10]. Quercetin (QC) is a bioflavonoid found abundantly in various fruits and vegetables. The suggested beneficial effects of QC include a range of health benefits, including protection against cardiovascular diseases [11], supporting neurological function [12], preventing gastrointestinal damage [13], inhibiting cancer growth [14], suppressing bacterial growth [15], reducing the risk of atherosclerosis [16], alleviating inflammation [17, 18], promoting antioxidant activity [17, 19], modulating the immune system [18], maintaining lipid balance [20], and preserving bone health [21]. Several biological activities exhibited by QC can potentially play a role in maintaining bone health [22].

Given the diverse therapeutic potential of QC, this review aims to provide an extensive analysis of the effects and underlying mechanisms of QC concerning osteoporosis, sarcopenia, and tendon-related conditions. Moreover, the study aims to clarify QC's regeneration properties and regulatory mechanisms in the musculoskeletal system. A detailed mechanistic understanding of the antioxidant properties of QC can facilitate the identification and development of novel targets to address musculoskeletal aging problems. Specifically, the role of QC in the catabolic processes and oxidative stress contributing to musculoskeletal damage and regeneration has been comprehensively reviewed.

## 2. QC

QC, also known as 3,3′,4′,5,7-pentahydroxyflavone, is a generally recognized flavonoid compound in the classified flavonol group [23]. This specific flavonoid is present in a diverse range of fruits, particularly citrus fruits, as well as seeds, leafy vegetables, trees, flowers, nuts, green tea, broccoli, onions, olive oil, apples, raisins, red wine, dark cherries, and berries such as blueberries and cranberries. As a result, it is abundantly available in our diet. Vegetables like onions and broccoli are recognized for their abundant QC contents and potent antioxidant properties. Similarly, cherries, apples, berries, red wine, and tea are recognized for their significant amount of QC. The substance is yellowish and has limited solubility in hot water. However, it dissolves completely in lipids and alcohol but remains insoluble in cold water. Numerous research initiatives are underway to enhance flavanols' bioavailability [24].

QC exhibits a diverse array of pharmacological properties, including its anti-cancer activity, ability to mitigate allergic reactions, anti-inflammatory effects, potential to manage diabetes, and potential to improve bone quality. Studies have been conducted regarding applying QC as a nutritional additive, indicating its potential effectiveness in addressing various diseases. In addition, QC serves as a potent antioxidant that triggers various physiological reactions within the human body, potentially alleviating anti-inflammation [25]. Table 1 summarizes the effects of QC on various physiological processes in bone, muscle, and tendon.

## 3. Role of QC in bone

Bone homeostasis refers to regulating a dynamic equilibrium between osteoblasts, responsible for bone synthesis, and osteoclasts, which cause bone degradation. A discrepancy in the bone remodeling procedure, where there is a higher rate of breakdown of osteocytes compared to bone formation driven by osteoblasts, results in a reduction in bone mineral density (BMD) and the elements of the bone matrix, such as bone marrow cells (BMCs) [26]. Osteocytes play a substantial role in synthesizing new bone that is more susceptible to fractures and joint deterioration in conditions like osteoporosis [27].

**Table 1:** Effects of QC on various processes associated with the musculoskeletal system.

Tissue	Process	Effects of QC	Reference
**Bone**	Osteoblastogenesis	Activates osteoblasts through the induction of mitogen-activated protein kinase (MAPK) pathway	[28]
	Osteoclastogenesis	Inhibits the expression of markers associated with osteoclast	[29]
**Muscle**	Muscle differentiation	Stimulates the movement of myoblasts via activating the integrin beta-1 (ITGB1) signaling pathway, which involves the phosphorylation of focal adhesion kinase (FAK) and paxillin	[30]
	Muscle regeneration	Stimulates migration and differentiation processes, which ultimately enhances muscle regeneration	[30]
	Muscle atrophy	Reduction in epididymal adipose tissue and augmentation in the mass of quadriceps and gastrocnemius muscular tissues	[31]
**Tendon**	Tendinopathy	Reduces the expression of nitric oxide (NO) and elicits anti-inflammatory effects	[32]
	Tendon adhesion	Exhibit inhibitory effects on oxidative stress	[33]

Understanding the probable mechanism of osteoporotic fracture is extremely important [34]. A difference in the production frequency of osteoblasts and osteoclasts leads to accelerated bone resorption compared to bone repair. In these situations, the bone loss rate surpasses the bone formation rate, resulting in a reduction in bone size, a health condition known as osteoporosis [35]. Osteoporosis is a medical disorder where the bone marrow space expands because of a gradual decline in BMD and a resulting drop in calcium content. Osteoporosis is a disease characterized by diminished bone strength, which increases the susceptibility of bones to fractures even with modest force exerted. This condition often manifests with symptoms like back pain, joint soreness, and fatigue. Current scientific research on osteoporosis treatment predominantly focuses on examining the balance, differentiation, and regulation mechanisms of osteoblasts and osteoclasts, which play crucial roles in bone creation and resorption [36]. The main therapeutic strategies for osteoporosis comprise the use of anti-absorbents, such as bisphosphonates, selective estrogen receptor modulators (SERMs), and anti-receptor activators of nuclear factor kappa-B ligand (RANKL) antibodies [37]. Several clinical trials have shown evidence supporting the efficacy of numerous osteoporosis treatments. Nevertheless, the continued use of compounds is linked with detrimental consequences that can endanger a person's life in old age. Hence, there is a pressing need to develop novel treatment approaches that may successfully address the issue of adverse effects in a substantial number of patients suffering from osteoporosis [38].

Recently, investigations on the role of flavonols in bone-forming bioactivity have given hope for an alternative potential drug treatment regime for age-related bone loss, like in osteoporosis. Studies have demonstrated that QC has various effects on different cell types of bone. It has been found to enhance programmed cell death in osteoblasts, stimulate angiogenesis, boost the production of antioxidants, and induce programmed cell death in adipocytes [39, 40]. Simultaneously, QC inhibits the formation of osteoblasts through RANKL, diminishes oxidative stress, safeguards osteoblasts from programmed cell death, and suppresses the inflammatory response. QC might utilize several potential mechanisms to exert its therapeutic effects, including the WNT and BMP signaling pathway, nuclear factor-kappa B (NF-κB) pathway, nuclear factor erythroid 2-related factor 2 (Nrf2) pathway, Smad dependence, and the regulation of intrinsic and extrinsic apoptotic pathways [41-43].

Experiments in animal models have also established the positive impacts of QC on bone health. However, a few studies have reported otherwise [44, 45]. The investigations have offered definite proof of the inhibitory properties of QC on RANKL-mediated oxidative stress, osteoclastogenesis, osteoblast apoptosis, and inflammatory response [46]. In addition, it has been found that QC has the potential to stimulate angiogenesis, osteogenesis, adipocyte apoptosis, the production of antioxidants, and osteoclast apoptosis. In contrast, previous studies have demonstrated that QC has complex and contradictory effects on the MAPK signaling pathway, vital for modulating bone metabolism [22].

### 3.1. Effect of QC on Osteoblasts

The WNT/β-catenin signaling system and the BMP/ transforming growth factor-beta (TGF-β) signaling primarily regulate the development and proliferation of osteoblasts [47]. The WNT signaling pathway is crucial in regulating skeletal development [30]. Three different WNT pathways have been discovered: the canonical pathway known as WNT/β-catenin and two non-canonical pathways called WNT/planar cell polarity (PCP) and WNT/Ca^2+^ pathways [48]. The Wnt family comprises crucial glycoproteins involved in various developmental processes and tissue maintenance. WNT5a, a member of the noncanonical WNT family, influences embryonic development through multiple signaling pathways. It activates β-catenin and binds to receptor tyrosine kinase-like orphan receptors (ROR), particularly ROR2, impacting cell functions and axis extension in embryos. The WNT5a-ROR2 pathway has a crucial function in development, making it a promising target for diagnosing and treating human disorders [49]. Jun kinase (JNK) is activated by the non-canonical PCP pathway, aided by disheveled (DVL) and the Rho small RAC and GTPases. The non-canonical WNT/Ca^2+^ pathway involves interacting with ROR, WNT, and Frizzled (FZD) receptors, releasing intracellular calcium ions and activating calcium-sensitive enzymes. Further, this specific event stimulates the nuclear factor of activated T cells (NF-AT), which subsequently translocate to the nucleus and initiate the transcription of specific genes. The WNT signaling pathways are also essential in bone formation since they regulate the differentiation of chondrocytes and osteoblasts [50].

The osteogenesis involves the participation of several transcription factors, including runt-related transcription factor 2 (Runx-2), collagen type 1 (Col1), and core-binding factor alpha 1 (Cbfα1) [51]. Furthermore, this physiological process is significantly influenced by vascular endothelial growth factor (VEGF) and bone biomarkers such as bone choline, bone sialoprotein (BSP), and alkaline phosphatase (ALP). These transcription factors are frequently used as crucial markers for evaluating the osteogenic ability of QC [22]. QC interacts with various bone components at a molecular level, impacting bone formation and resorption [52]. QC can increase gene expression levels of Runx2 and Osterix (SP7). QC attenuates oxidative stress injury via the Nrf2/HO-1 pathway in ferric ammonium citrate treated-MC3T3-E1 cells. QC rescues the dysfunction of osteogenic differentiation by preventing inhibition of proliferation and reduction of Runx2 and SP7 expression in ferric ammonium citrate treated-MC3T3-E1 cells [53]. QC can stimulate osteoblasts' osteogenic abilities by activating the MAPK pathway [28]. Adding QC 3-glucuronide at a dose of 5 μM to rat osteoblast-like ROS 17/2.8 cells resulted in an upregulation of Cbfα1/Runx-2 and BSP gene expression [54]. At doses of either 10 or 100 μM, QC-glucoside resulted in a surge in ALP activity, mineralization, osteocalcin, Runx-2, BMP2, and Col1 synthesis. When a concentration of 20 μM of QC aglycone was added, there was an increase in mineralized nodules, Runx-2 expression, ALP activity, BSP production, and osteocalcin levels in osteoblasts obtained from rat calvaria at the embryonic stage that were exposed to hydrogen peroxide (H_2_O_2_) [55]. The phenomenon observed in human osteoblast-like SaOS-2 cells involved the upregulation of Runx-2, its co-activator ATF6, and Ets1 upon exposure to QC 3-β-D-glucoside and polyphosphoric acid. When exposed to QC, the murine osteoblasts MC3T3-E1 showed more significant cellular proliferation, increased expression of BSP, enhanced ALP activity, better osteocalcin production, increased collagen synthesis, and improved mineralization [56]. Prior research has shown that QC can increase the protein levels of β-catenin and WNT3 in osteoblasts. Pre-treating with QC has been reported to counteract the apoptosis caused by lipopolysaccharide (LPS) and prevent the osteogenic effects on MC3T3-E1 cells. The inhibitory effect of QC's protective characteristics was exhibited following prior administration of the MAPK inhibitor or the WNT/β-catenin inhibitor XAV939 [57]. Prior research has demonstrated that QC effectively safeguards against tumor necrosis factor α (TNF-α) inhibitory effects on osteoblast [58]. The protective mechanism involves silencing NF-γB (nuclear factor-γB) and the breakdown of β-catenin in rat bone marrow stromal cells.

Furthermore, it has been shown that the presence of estrogen-like QC chemicals might improve the process of bone formation in bone marrow mesenchymal stem cells (BMSCs). The validation of estrogen signaling was carried out by exposing BMSCs to ICI1827280, a selective inhibitor that targets the endoplasmic reticulum. Administration of ICI182780 reduced the levels of BMP2, Smad1, Smad4, and p-Smad1. This emphasizes the significance of QC in promoting the differentiation of BMSCs by activating the BMP and estrogen receptor (ER) signaling pathways [59]. The findings reported by Kim et al. indicate that the osteoblast differentiation in human periodontal ligament cells can be achieved by activating BMP signaling, as well as the WNTβ-catenin and MAPK signalings, using Myricetin, BMP2, and Smad1/5/9. Elevated phosphorylation of BMP receptor IB was observed, leading to the subsequent activation of osteogenesis-related proteins, specifically Runx-2 and osterix [56]. Furthermore, empirical data suggests that Isocercetin can boost the proliferation of BMSCs by activating BMP signaling [60].

Yuan et al. reported that QC can protect against the inhibition of osteoblast differentiation by TNF-α on rat BMSCs. The researchers witnessed the stimulation of NF-κB and the degradation of β-catenin in cells that were subjected to TNF-α. However, these effects were counteracted when TNF-α-treated cells were subsequently treated with QC [58]. A preclinical study found that ovariectomy downregulated the level of β-catenin in the femurs of rats. In contrast, gene expression levels of NF-κB and HIF-1α increased. A supplementation with isoQC in rats restored the expression of HIF-1α and β-catenin and improved bone quality in rats [61]. QC targets the H19/miR-625-5p axis to activate the WNT/β-catenin pathway, promoting BMSC proliferation and osteogenic differentiation [62]. QC prevents oxidative stress by increasing gene expression of HO-1 in BMSCs [63]. QC reduces ROS levels by upregulating antioxidant genes such as catalase (CAT), SOD-1, SOD-2, and Nrf2 in BMSCs [64].

### 3.2. Effect of QC on osteoclasts

Multiple investigations have provided insights into how QC affects osteoclastogenesis and its ability to inhibit the expression of markers associated with osteoclast formation in RAW264.7 cells. These cells are derived from unicellular/macrophage and osteocyte precursor cells in mice [65]. Bone marrow cells from mice, when exposed to parathyroid hormone (PTH), which is known to promote bone resorption, increased the development of cells that resemble osteoclasts. However, this rise was inhibited when QC (at concentrations ranging from 0.01 to 1 μM) was administered [66]. Through various regulatory signaling pathways, including WNT/β-catenin, BMP/SMAD/Runx-2, and OPG/RANKL/RANK, QC exerts beneficial effects on bone remodeling by reducing osteoclastogenesis [52]. QC-functionalized hydroxyapatite (HA) showed promising effects on bone repair by downregulating osteoclastogenesis, promoting osteoblast proliferation and differentiation, and supporting endothelial cell functions. The study also suggested that quercetin may have potential therapeutic benefits for enhancing bone regeneration and mitigating bone loss conditions when incorporated into biomaterials for local administration in bone repair applications [67]. Likewise, various analogs of QC, such as QC 3-O-β-D-glucuronide, QC-6-C-β-d-glucopyranoside, and QC, reduced the number of TRAP-positive cells and the area of bone resorption pits while RANKL induced osteoclast differentiation in both RAW264.7 cells and bone-marrow macrophages and bone-marrow macrophages [68-70]. QC, administered at 150 mg/kg either alone or in combination with alendronate, effectively prevented glucocorticoid-induced osteoporosis (GIO) in Sprague-Dawley rats. This protective effect was demonstrated as a notable augmentation in femur-breaking strength and bone thickness compared to methylprednisolone-treated rats. Moreover, QC notably elevated osteocalcin levels, indicating its potential as a stimulant for bone growth, but alendronate failed to exhibit this effect. These findings suggest that QC, particularly at the mentioned dosage, holds promise as a preventive measure against GIO, potentially outperforming alendronate in some aspects [71]. QC-3-O-β-D-glucuronide suppresses JNK and ERK activation in LPS-stimulated RAW264.7 macrophages and inhibits expressions of NO and PGE secretion and inducible nitric oxide synthase (iNOS) and cyclooxygenase-2 (COX-2) expression [72]. QC exerts anti-apoptotic effects through JNK-c-Jun/AP-1 and ERK-c-Fos/AP-1 pathways [73]. QC significantly reduces TNF-α and interleukin (IL-6) levels in LPS-induced mouse RAW264.7 macrophages [74]. QC inhibits M1 polarization and reduces M1 markers such as IL-6, TNF-α, and IL-1β in macrophages and microglia [75]. QC reduces TNF-α and IL-1β levels, inhibiting the activation of osteoclast and attenuating bone destruction [29].

## 4. Role of QC in skeletal muscle

The process of aging and the presence of muscular problems often result in a decrease in the locomotion and differentiation of myoblasts, ultimately leading to impairments in the functioning and ability to regenerate skeletal muscles. Numerous studies have provided scientific evidence that supports the claim that natural flavonoids can stimulate muscle hypertrophy [76-78].

### 4.1. Myogenesis and regeneration

Myogenesis refers to the biological process by which skeletal muscle tissue is formed. This mechanism predominantly occurs during the embryonic stage of development. During myogenesis, mononucleated myoblasts, depicted as violet, merge to create multinucleated muscle fibers, distinguished by their multiple nuclei. Muscle fibers are typically formed by merging precursor myoblasts, forming multinucleated fibers known as myotubes. During the initial phases of embryonic development, myoblasts possess the ability to either divide or undergo a process called differentiation, leading to the formation of myotubes. The first phase entails the cessation of the biological cycle and the initiation of gene expression tailored to particular conditions. The following cellular differentiation phase involves the myoblasts' arrangement and positioning. The research findings indicate a significant amount of data that supports the identification and arrangement of myoblasts in rats and chicks. This suggests that evolutionary processes may have preserved the fundamental mechanisms responsible for this phenomenon. The subsequent phase is cellular fusion. Calcium ions are essential throughout this period [79]. Fusion in humans is enabled by a group of metalloproteinases encoded by the ADAM12 gene and other proteins. The fusion process entails binding actin proteins to the plasma membrane, followed by the precise alignment of membranes and the consequent development of a rapidly expanding pore. Myogenesis can be classified into many discrete phases. In embryonic myogenesis, structures derived from the mesoderm play a vital role in the initial development of the basic muscle fibers in the organism. As a result, muscle fibers are further formed in succeeding waves, following the established pattern provided by the original template fibers [80]. There is a lack of understanding about the behavior of muscle-specific myogenic progenitor cells throughout the perinatal phase. At first, these progenitor cells undergo substantial proliferation. However, as the myonuclei number stabilizes and the production of myofibrillar protein reaches its maximum level, the population of myogenic progenitors gradually decreases [81].

After achieving full maturity, the indicated parental organisms will enter a stage of inactivity called quiescence, where they will establish their presence inside the muscle as satellite cells. Like other regenerative tissues, adult skeletal muscle depends on a system that enables the replacement of mature cells, hence maintaining the stability of the tissue [82]. There are many similarities between the process of muscle development in embryos and the process of muscle regeneration in fully mature skeletal muscle. These similarities include the presence of similar transcription factors and signaling molecules. Scientists widely agree that satellite cells are robustly associated with progenitor cells that emerge from the somites [83-85]. The interaction of QC with skeletal muscle components at the molecular levels has attracted considerable interest owing to its potential impact on muscle performance, recovery, and overall physiological health. QC scavenges free radicals, which can damage the myoblasts, and chelates metal ions that produce ROS, reducing oxidative stress and protecting muscle cell integrity [19]. QC can promote migration and differentiation processes, improving muscle regeneration. A recent study described the observed phenomenon of the anti-atrophic action of QC on muscle cells. Phosphorylation of the insulin-like growth factor 1 receptor (p-IGF-1R) in murine C2C12 cells increased migration and muscle differentiation during the initial growth phases. QC's regulatory capability to impact the ITGB1 signaling pathway increases myoblast migration, activating FAK and paxillin [30]. In addition, myoblast differentiation is characterized by the activation of transcription factors STAT3 and the AKT signaling pathway [30].

### 4.2. Muscle atrophy and sarcopenia

Muscle atrophy, i.e., muscle wasting, is a detrimental condition that affects skeletal muscle. It is characterized by decreased muscle mass, area, and functionality [86]. Muscle atrophy not only leads to a decrease in muscular function but also hampers the effectiveness of therapies, consequently affecting the overall standard of life and exacerbating both mortality and morbidity rates [87]. Chronic disorders, including cancer, chronic obstructive pulmonary disease (COPD), diabetes, and obesity, as well as the natural process of aging and situations of inactivity, such as immobility, bed rest, and mechanical unloading, are the primary causes of muscle atrophy [88]. Furthermore, muscle atrophy can be ascribed to several intrinsic reasons, such as genetic susceptibility, hormone imbalances, and the physiological repercussions of aging [86, 89]. In addition, muscle atrophy can also be attributed to different environmental variables such as stress, injury, and lack of usage [90]. Cachexia is the term used to describe the pathological condition when muscle tissue degenerates due to a disease. Sarcopenia is the medical phrase that describes the decline in strength and muscle mass that occurs with advancing age [91]. Sarcopenia is a medical condition marked by the gradual reduction of muscle mass and deterioration of physical and/or strength capacities that occur with advancing age. This syndrome has significant health hazards, including increased susceptibility to various diseases and higher death rates among the older population [92]. The term "sarcopenia" was coined by Irvin Rosenberg [93] in 1989 to describe the decrease in skeletal muscle mass, strength, and function that occurs with aging. This condition has emerged as a significant global health issue, mainly due to the rapid increase of the older population globally [94]. The aetiology of various muscular disorders, including sarcopenia, cachexia, and disuse muscle atrophy, may differ, but they all lead to similar harmful effects. These effects encompass oxidative stress, disruption in protein breakdown and synthesis, inflammation, and mitochondrial dysfunction [95-97]. These reasons exemplify a symbiotic or interdependent connection [96, 98]. Family medicine and internal medicine have led in defining, studying, and developing treatments for sarcopenia, with a particular focus on diabetes and metabolic diseases. Hence, the use of existing diagnostic criteria for sarcopenia, which range throughout continents, presents difficulties in evaluating orthopedic patients with hip fractures [99].

Comprehensive research has been carried out to examine the potential of QC in mitigating wasting conditions in several experimental models related to obesity, muscle atrophy, muscular diseases, and cachexia. The significant effect of QC on reducing obesity has been demonstrated in various experimental animal studies [100, 101]. As a result of this phenomenon, numerous research has investigated the impact of QC on muscle wasting in obese mice. The administration of QC led to decreased epididymal fat and a rise in the weight of the quadriceps and gastrocnemius muscle tissues in mice that were made obese by a high-fat diet. The results indicate that QC possesses properties that can counteract obesity and mitigate muscular atrophy [31].

QC was proven to induce a reduction in the production of MuRF-1 and atrogin-1 in the gastrocnemius muscle of obese mice. In addition, QC reduced the number of transcripts that encode monocyte chemoattractant protein-1 (MCP-1) TNF-α. In addition, the study found that exposing RAW264.7 macrophages to palmitic acid reduced the levels of MuRF-1 mRNA and atrogin-1 in C2C12 myotubes. Kim et al. showed that the decrease in the diameter of myotubes produced by TNF-α was seen after the administration of QC. The reported effect was achieved by suppressing both atrogin-1 in C2C12 myotubes and the mRNA and protein levels of MuRF1 in vitro and obese mice. QC protects against muscle wasting caused by TNF-α in the context of obesity. The indicated protective mechanism activates Nrf2, which increases heme oxygenase-1 (HO-1) gene expression. Moreover, QC impedes the activation of NF-κB [102]. QC can enhance mitochondrial biogenesis while forming new mitochondria in cells and is crucial for improving muscle endurance and energy production. QC activates AMPK and sirtuin 1 (SIRT1), which subsequently upregulates PGC-1α. PGC-1α is a crucial regulator of mitochondrial biogenesis [103-105]. Also, QC reduces mitochondrial dysfunction by activating AMPK/SIRT1 signaling pathway in the osteoarthritis rat model [106]. Additionally, exercise induces inflammation in skeletal muscle, which can be detrimental if prolonged [107]. QC can modulate this response by inhibiting NF-κB and COX-2 and suppressing pro-inflammatory cytokines like TNF-α and IL-6 [108, 109]. QC inhibits the ubiquitin-proteasome pathway and reduces protein degradation via the mTOR pathway (mTOR/4EBP1 phosphorylation). Therefore, QC plays a crucial role in muscle hypertrophy (growth) and atrophy (wasting) by regulating the balance between protein synthesis and degradation [110]. Maintaining calcium homeostasis is essential for skeletal muscle contraction. QC regulates the release and reuptake of calcium ions by interacting with the sarcoplasmic reticulum of muscle cells. Maintaining calcium homeostasis is vital for skeletal muscle contraction. QC regulates the release and reuptake of calcium ions by interacting with the sarcoplasmic reticulum of muscle cells [111]. QC can promote myogenic differentiation, MHC expression, and muscle tissue repair and regeneration by affecting GSK-3β/β-catenin, AKT/4EBP1, and phosphorylation of STAT3 [30].

An investigation was conducted on the impact of QC on muscle atrophy in animal models. The atrophy was produced either by clinorotation or dexamethasone [112]. During the experiment, it was noticed that QC hindered the production of MuRF1 and atrogin in C2C12 myotubes in clinorotation conditions by inhibiting the phosphorylation of ERK. However, it was demonstrated that QC did not contribute to reducing gene expression in C2C12 cells treated with dexamethasone [113]. The ensuing finding contradicts a prior investigation showcasing QC glycoside's preventive properties against muscle atrophy generated by dexamethasone in mice and C2C12 myotubes. Following the administration of QC glycoside, the gene expression levels of MurF1 and atrogin-1 appeared to decline in C2C12 myotubes that were dexamethasone-treated. When mice treated with dexamethasone were given QC glycoside, there was an increase in the ratio of gastrocnemius muscle weight to body weight. The observed outcome was accomplished by reducing the expression of atrogin-1, MuRF1, and myostatin mRNA.The observed inconsistencies arise from variations in the models, dosages, or structural differences between QC and QC glycosides. Under typical circumstances, QC produces favorable impacts on muscle tissues. QC treatment stimulated mitochondrial biogenesis and improved the ability to tolerate exercise in mice via activating SIRT1 and PGC-1α [114]. The administration of QC in C2C12 myotubes increased transcriptional activity at the TFAM promoter [115]. Numerous scientific studies have thoroughly demonstrated the therapeutic effectiveness of QC, including its ability to reduce oxidative stress and inflammation and promote muscle health. These qualities have been found to alleviate muscular atrophy, as evidenced by many studies [116]. The QC administration effectively inhibited the transmission of signals linked to muscle deterioration, promoting muscle atrophy in mice subjected to immobilization or glucocorticoid treatment [113, 117].

## 5. Role of QC in tendon

Tendon diseases cover a spectrum of conditions, ranging from persistent tissue deterioration to sudden accidents resulting in partial or complete tendon rupture. Tendon injuries can disturb the delicate equilibrium between stability and mobility, resulting in reduced physiological functioning and the development of various disabilities. The Achilles tendon is the most often seen location of rupture in the human body. Age-related alterations in the cells as well as the extracellular matrix (ECM) of tendons and ligaments, especially those present in the knee joints, including the anterior cruciate ligament (ACL), are some of the pivotal factors responsible for causing osteoarthritis (OA) [118, 119].

The injury in the tendon and its nearby tissues produces a significant variety of growth factors and cytokines. The substances included in this category include interleukins (IL), TNF, platelet-derived growth factor (PDGF), and vascular endothelial growth factor (VEGF). Fibroblast growth factor (FGF), transforming growth factor-beta (TGF-β), epidermal growth factor (EGF), connective tissue growth factor (CTGF), and insulin-like growth factor (IGF)-1 are some examples of growth factors commonly studied. The initial inflammatory phase following tendon injuries is characterized by the production of TNF and IL, which are mainly released by the M1 macrophages [120, 121]. In contrast, the secondary inflammatory response is caused due to the anti-inflammatory M2 macrophages. Macrophages are essential in facilitating neovascularization. This is achieved by the secretion of several growth factors. In addition, they play an active role in developing fibrous tissue by secreting profibrotic chemicals such as CTGF and TGF-β. Furthermore, animal models have shown that tendon injury causes a substantial rise in mRNA levels for various genes involved in collagen synthesis (Col1a1, Col1a2, Col3a1, Col12a1, Col14a1), as well as substances associated with tendons such as proteoglycans, tenomodulin (Tnmd), and tenascin (Tnc) [122, 123]. The overexpression of the ECM gene in animal models of tendon damage is also notably found in various cases of human tendinopathy. Following tendon injury, there is an apparent upregulation of genes that encode transcription factors related to tendons, namely Mohawk (Mkx), scleraxis (Scx), and EGR1 (Egr1) [120, 122, 123].

Tendon healing after surgery can be subdivided into three categories: an initial phase of inflammation lasting about one week, followed by a period of cell growth and multiplication lasting several weeks, and finally, a phase of tissue remodeling that extends over several months [124]. Chronic tendon injury is the most common damage caused by the overuse of tendons. It is marked by the existence of discomfort along with diminished ability and effectiveness of the tendons [125]. Tendinopathy is marked by the presence of pain and reduced physical ability. The cause of tendinopathy is not entirely understood and is often characterized as either a degenerative disorder or a weakness in the body's reparative mechanisms. Furthermore, cases of overuse tendinopathy have been clinically reported to exhibit elevated levels of COX-2 and IL-6. It is understood that the production of these inflammatory cytokines does not adhere to the traditional description of an inflammatory response. Alternatively, it has been postulated that resident tenocytes show this phenomenon as a response to severe mechanical stress. From a histological standpoint, one may witness the disruption of collagen structure, elevated quantities of non-collagenous ECM, increased cell density, and the formation of various blood vessels [126, 127].

Several research studies have elucidated scientific evidence about natural flavonoids, claiming they positively affect tendocytes.

QC interacts with various components of tendons at a molecular level, influencing tendon health, repair, and function. Tendons must withstand mechanical stresses and strains during movement. QC increases the tensile strength and elasticity of the tendon by promoting the cross-linking of collagen fibers and maintaining the essential biomechanical tendon's function by improving collagen synthesis, proteoglycan synthesis, and ECM regulation (inhibiting MMPs and aggrecanases), resulting in enhancing the tendon's mechanical properties [33, 128-130]. ICAM-1, STAT3, and MMPs are involved during tendinopathy. QC reduced ICAM-1 and MMP expression in tendon tissue [129]. QC is critical for fibroblast function and ECM production by modulating the TGF-β signaling pathway [131]. Tendons easily damage their function and healing because they are susceptible to oxidative stress. QC neutralizes ROS, reducing oxidative damage to tendon cells and ECM components. In addition, QC protects tendon cells against oxidative stress by upregulating the activity of antioxidant enzymes like SOD and CAT [32]. QC suppressed pro-inflammatory cytokines such as TNF-α, IL-1β, IL-6, and NF-κB signaling pathways involved in tendon inflammation and degradation [129, 132].

QC treatment decreases NO levels and subsequently triggers anti-inflammatory and antioxidative effects in the Achilles tendons of diabetic rats [32]. QC showed promise in its ability to demonstrate efficacy in treating diabetic tendinopathy. Recent research studies have extensively shown the inhibitory effects of QC on oxidative stress and its ability to minimize tendon adhesion in rats after transection and healing. This occurrence is linked to increased amounts of glutathione peroxidase (GPx) and SOD, along with decreased levels of malondialdehyde (MDA). Giving QC can raise GPx and SOD levels while also lowering MDA levels in a way that depends on the dosage [33]. In addition, a comparative study showed that the high QC dose treated group had a somewhat decreased level of tendon adhesion compared to the low QC dose treated group and the control. Also, the histological examination of the significant organs revealed no apparent signs of toxicity with QC [133, 134].

Administering collagenase injection triggers a series of biological reactions, including inflammation, apoptosis, oxidative stress, and autophagy. Furthermore, the involvement of MMPs, ICAM-1, signal transducer, and STAT3 has been reported in the pathogenesis of tendinopathy. Administration of QC decreased the levels of ICAM-1 in tendon tissue. Furthermore, the analysis of QC data revealed that QC has properties that counter inflammation, oxidative stress, apoptosis, and autophagy, especially with tendons. This implies that QC might offer therapeutic advantages in treating tendinopathy. Furthermore, studies highlighted the role of QC in inhibiting the expression of MMPs in tendon tissues. Hence, it may be argued that QC can be a viable treatment methodology for athletes or others afflicted with tendon injuries [134].

## 6. Antioxidant activity of QC for bone, muscle, and tendon regeneration

Oxidative stress refers to a condition that occurs due to the generation of ROS and their removal from an organism's extracellular or intracellular systems. ROS are comprised of the superoxide anion, hydroxyl radical (OH), radical (O_2_-), H_2_O_2_, and singlet molecular oxygen (O_2_). The superoxide anion reacts with NO and hydro H2O2 gen peroxide (or hydrop-eroxyl) radicals (OOH) under acidic conditions, resulting in the formation of reactive nitrogen peroxide (ONOO-). GPx facilitates the enzymatic conversion of H_2_O_2_ peroxide into water. The production of H_2_O_2_ is solely responsible for the formation of very reactive OH, which is enabled by transferring a single electron from transition metal ions (known as the Fenton reaction). Hence, it is clear that the removal of O_2_- and other reactive species in the human body is enhanced by the combined efforts of two antioxidant enzymes, namely SOD and GPx [19].

Oxidative stress can occur due to the disparity between generating and removing reactive nitrogen species (RNS) and ROS [135]. ROS are generated internally by several events involved in cellular metabolism. RNS are chemical compounds arising from the interaction between NO and other substances, including ROS and RNS, which may be categorized into two classes based on their chemical properties: radical or non-radical molecules. The radical ROS comprises the O_2_- and HO, whereas the non-radical ROS comprises H_2_O_2_. RNA contain NO and ONOO- [136].

Flavonoids have a wide range of pathways by which they can effectively ameliorate circumstances of oxidative stress. These mechanisms include scavenging ROS, enhancing the activity of antioxidant enzymes, binding with metal ions, decreasing the activity of oxidases and α-tocopheryl radicals, reducing oxidative stress caused by high levels of uric acid and NO, and enhancing the antioxidant properties of low-molecular-weight antioxidants. Flavonoids also exhibit pro-oxidant properties and facilitate the oxidation of other substances [8].

### 6.1. Role of QC in bone during oxidative stress condition

In a laboratory-based experimental study, the researchers observed that the antioxidant capabilities of QC and its related glycosides are responsible for their osteoblastic and anti-osteogenic activities [137]. The osteogenic potential of QC was further investigated in cellular models subjected to inflammatory cytokines, menadione, LPS, H_2_O_2_, and components of tobacco smoke [138]. QC efficiently mitigated the adverse effects of TNF-α on osteogenic disorders in rat BMSCs and MC3T3-E1 murine progenitor cells [22]. QC counteracted the inhibition caused by LPS stimulation on the development and maturation of osteoblasts. This was achieved by enhancing the activity of genes specific to osteoblasts, including ALP, Runx-2, osteocalcin, BSP, etc [28]. Exposure of primary human osteoblasts to vehicles containing tobacco smoke led to rapid production of ROS, which in turn caused a decrease in cellular viability. Treatment with QC resulted in the reversal of the apparent effect, leading to an increase in the level of SOD and HO-1 [138]. This protective effect was observed because of increased cell viability. An additional study has shown that the concurrent growth of osteoblasts and osteoclasts, together with the application of H_2_O_2_, resulted in the generation of ROS. When co-cultures were exposed to HA supplemented with QC in the presence of H_2_O_2_, a decrease in ROS levels was observed. Studies elucidated QC’s protective effect from oxidative stress caused by H_2_O_2_ in osteoblasts. This defense system reduces ROS and protects important biological components, namely MDA, nitrotyrosine, and protein carbonyls [139].

Messer et al. conducted research where osteoblasts obtained from embryonic rat calvaria were treated with QC aglycone. The authors observed significant regulation of CAT, HO-1, glutamate-cysteine ligase catalytic subunit (GCLC), and peroxiredoxin-5 (Prdx5) in these osteoblasts [140]. A group of scientists in another study harvested osteoblasts from fetal mice. Osteoblasts were exposed to H_2_O_2_, which caused oxidative stress, and then were treated with QC aglycone [137]. Without treatment, the osteoblasts consistently showed increased expression of HO-1 and GCLC. Nevertheless, the introduction of QC aglycone hindered the process of up-regulation. QC enhanced the protection of primary human osteoblasts against oxidative stress by activating the ERK and Nrf2 signaling pathways. Treating osteoblasts with QC resulted in the phosphorylation of ERK1/2 and Nrf2 [141]. A similar effect of the administration of QC has been suggested with a reduction in oxidative and nitrifying stress levels in osteocytes [141]. According to Whatel et al., the introduction of QC downregulated ROS production in isolated rabbit bone cells [142]. Tang et al. conducted a subsequent investigation and showed that the use of QC decreased the levels of NO and ROS production stimulated by LPS in RAW264.7 cells. QC treatment resulted in an apparent reduction in the production of iNOS in RAW264.7 cells that were subjected to macrophage colony-stimulating factor (M-CSF) and receptor activator of RANKL [135].

**Figure 1.**
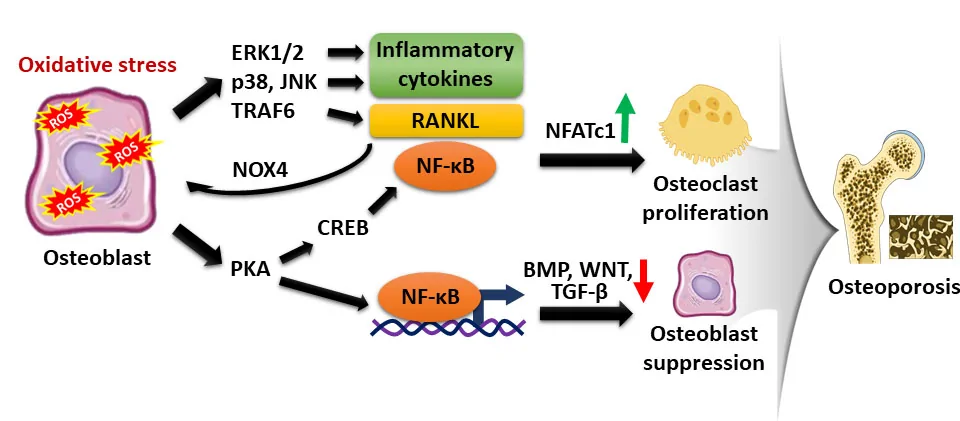
**Increased oxidative stress leads to bone loss**. Oxidative stress leads to osteoporosis by activating ERK1/2, p38, and JNK, leading to a release of inflammatory cytokines. TRAF6 then prompts RANKL production, initiating NF-ĸB activation and subsequently generating NFATc1. This cascade results in the excessive proliferation of osteoclasts, leading to progressive bone loss. Moreover, RANKL in osteoclasts activates NO4, yielding ROS. ROS also activates NF-ĸB via PKA pathways while impeding BMP, WNT, and TGF-β pathways. Consequently, inhibiting BMP, WNT, and TGF-β suppresses the differentiation of osteoblasts, leading to low bone formation. Increased bone loss accompanied by low bone formation leads to bone diseases like osteoporosis.

Several investigations undertaken on humans have provided actual evidence supporting the preventative effects of oxidative stress and the bone-preserving properties of QC. Research also proved that the levels of MDA decreased, and the reduced glutathione (GSH) content increased in rats that had undergone ovariectomy and in a bone loss model produced by retinoic acid. This was observed after administering unbound QC and QC-containing phytosome nanoparticles orally. The research results showed that the levels of 8-OHdG in the urine of rats with streptozotocin (STZ)-induced diabetes decreased after being given oral dosages of QC for eight weeks. In addition, the entire antioxidative potential, CAT, GPx, SOD, and glutathione S-transferase (GST) were elevated after administering QC [33]. T The mechanism by which oxidative stress leads to the release of inflammatory cytokines and subsequent proliferation of osteoclast along with the suppression of osteoblast is illustrated in Figure 1. Furthermore, it has been shown that when fetal rat calvaria-derived osteoblasts were exposed to QC aglycone, there was a significant increase in the expression of HO-1, CAT, GCLC, and Prdx5 [143].

### 6.2. Role of QC in skeletal muscle on the oxidative stress condition

Administering QC directly into the gastrointestinal muscle has demonstrated positive outcomes in lowering oxidative stress in animals subjected to unloading conditions. Furthermore, it was noted that QC inhibited the MuRF-1 and atrogin-1 in the gastrointestinal muscle [144]. Ingesting QC for a brief duration resulted in the upregulation of genes which are primarily responsible for mitochondrial biogenesis. Catechins, a kind of dietary antioxidants included in tea and chocolate, were reported to impede the production of ubiquitin-linked enzymes [145]. The study found that tea catechins, namely epigallocatechin gallate (EGCG), increased oxidative stress in the muscular fibers of rat muscles that had atrophy from unloading. In addition, the presence of tea catechins reduced the formation of protein carbonyls in these muscle fibers. EGCG treatment effectively suppressed mitochondrial depletion in the skeletal muscle of Goto-Kakizaki rats with diabetes [146]. The use of tea catechins substantially prevented the decrease in physical performance, as seen by improved mitochondrial activity in the skeletal muscle of mice [147]. Additionally, it was noted that EGCG effectively suppressed protein carbonylation in the skeletal muscle of rats [148].

The findings indicate that antioxidant flavonoids can reduce oxidative stress and, thus, potentially prevent DNA damage. The mechanisms that protect against muscle atrophy induced by unloading pertain to eliminating ROS inside mitochondria and/or regulating the cellular redox state. These processes subsequently resulted in the activation or suppression of signaling pathways. Le et al. demonstrated that administering QC to mice that were rendered obese by a high-fat diet reduced adrenal fat, followed by an increase in the weight of the quadriceps and gastrointestinal muscles. The results suggest that QC possesses anti-obesity properties and can alleviate muscular atrophy [31]. Following QC treatment, the levels of MuRF1 and atrogin-1 were found to decrease, and the quantity of MCP-1 and TNF-α in the gastrointestinal muscle of obese mice diminished. The study also added that the mRNA levels of MuRF1 and atrogin-1 decreased in C2C12 myotubes when exposed to palmitic acid [149]. Kim et al. demonstrated that QC had an antiatrophy effect in obese rats. They discovered that administration of QC inhibited the reduction of myotube diameter by TNF-α. The observed behavior was attained by reducing the protein levels and mRNA expression of MuRF-1 and atrogin-1 in C2C12 myotubes. This was ascribed to the activation of Nrf-2, which suppressed the NF-γB pathway and stimulated HO-1 [150]. The administration of QC reduced MuRF-1 protein in the muscular tissues of obese mice. The observed effects were achieved by manipulating the amounts of NF-γB protein and increasing the expression of HO-1 and Nrf-2 proteins [31].

**Figure 2.**
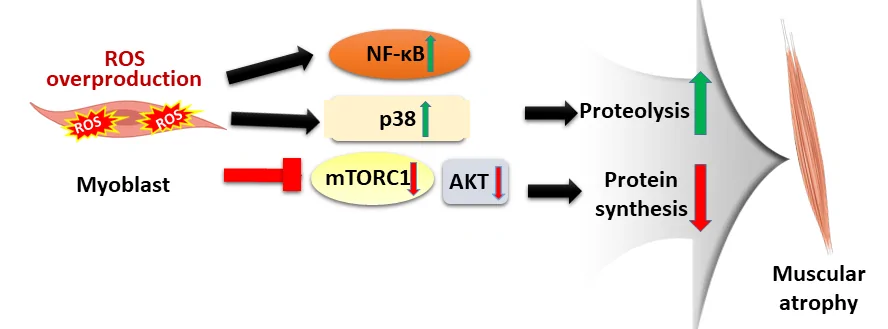
**Increased ROS production leads to muscular atrophy**. As muscles age, there is an excessive production of various ROS species. The elevated levels of ROS suppress the AKT/mTORc1 pathway, consequently restricting protein synthesis, which is a contributing factor to the onset of muscular atrophy. Moreover, the excessive ROS activates NF-ĸB and p38, facilitating proteolysis, leading to muscular atrophy.

Treatment of QC decreased IL-6 in the plasma of ApcMin/+ mice [151]. These mice serve as a model for colorectal cancer characterized by hyperemia, which refers to an abnormal increase in blood flow [152]. Consequently, QC led to a reduction in the IL-6/STAT3 signaling pathway. QC led to the overall increase in both body weight and the size of multiple anatomical components, including epididymal fat, gastrointestinal muscles, and quadriceps. Furthermore, the administration of QC to ApcMin/+ mice significantly increased grip strength compared to control. However, there were no observable differences in muscle durability [151]. When A549 lung cancer cells were surgically implanted in mice, it was shown that using QC improved the anti-cancer effects of trichostatin A [140]. The observed increase was identified by a reduction in tumor volume, which was ascribed to the overexpression of the p53 protein. An enhanced recuperation in animals with tumors and compromised gastrointestinal muscle function following the administration of trichostatin A was observed. The treatment involved including a diet supplemented with QC and delivery by intraperitoneal injection [153]. QC regulates protein synthesis for MuRF1 and atrogin-1 at the molecular level by sequestering FoxO1 in the cytoplasm [154]. Furthermore, QC therapy resulted in decreased lipid peroxidation levels and a drop in the amounts of TNF-α and IL-1β in both plasma and gastrointestinal muscle. The observed benefits is due to the anti-inflammatory and antioxidant properties of QC [155].

Aging leads to the continuous production of ROS, which in turn triggers the constant activation of particular intracellular signaling pathways, namely AKT/mTOR, as well as the involvement of p38 and NF-κB. This ongoing process leads to the deterioration of skeletal muscle tissue (Fig. 2). Another study assessed the impact of QC and flavones on muscular atrophy caused by inactivity in mice subjected to tail suspension. Flavones exhibited a modest rise in the weight of gastrointestinal muscles. Still, this impact was not statistically significant and did not match the extent of healing seen in the QC treatment group. Nevertheless, the addition of QC to the gastrointestinal muscles significantly augments the individual's total body weight. QC treatment led to a significant decrease in the expression levels of MuRF1 and atrogin-1 and a reduction in the quantity of thiobarbituric acid reactive compounds (TBARS) [156]. The study's findings have determined that the hydroxyl group of QC (QC-3-O-β-D-glucoside) is vital in reducing muscle atrophy caused by tail attachment. QC effectively slowed the progression of muscular degeneration induced by enucleation. This was demonstrated by a considerable increase in the mass of the gastrointestinal muscles [144]. The findings of a scientific investigation examining muscular atrophy due to tail suspension revealed that the introduction of QC reduced the expression of MuRF1. However, QC did not impact the mRNA expression levels of MuRF1 in mice subjected to inhibition [117]. Conversely, the levels of p-IGF-1, AKT, and PGC-1α showed a significant increase. The study demonstrated that QC treatment facilitated the restoration of muscle mass in injured muscles and increased the innate regeneration of neurons following nerve damage in the hind limbs of mice [157].

### 6.3. Role of QC in the tendon on the oxidative stress condition

**Figure 3.**
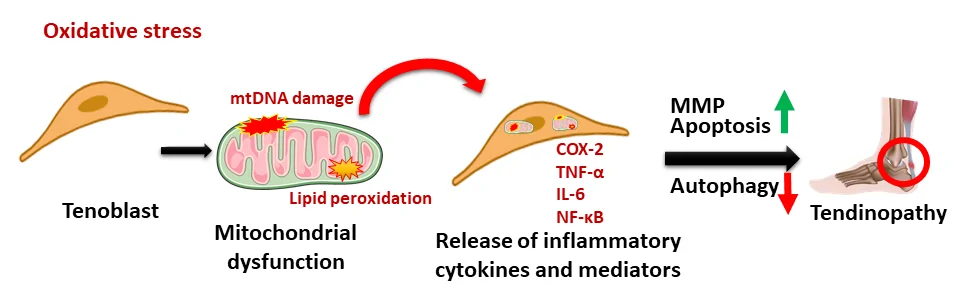
**Increased ROS production leads to tendinopathy**. Oxidative stress causes mtDNA damage and lipid peroxidation. Mitochondrial damage leads to the production of several inflammatory mediators and cytokines like COX-2, NF-ĸB, and IL-6. This induces tendinopathy by increasing apoptosis, MMP expression and inhibiting autophagy.

A delicate balance between ROS generation and the protective actions of antioxidants characterizes the process of turning nutrients into energy. However, the presence of oxidative stress disrupts this condition of balance. The association between the emergence of many degenerative and chronic diseases and a rise in oxidative stress in the human body has previously been demonstrated [158]. Tendinopathy is a prevalent musculoskeletal disease affecting athletes and adults aged 60 and beyond. Several risk factors, including repetitive activities, individual biomechanics, tendon structure, chronological age, and genetic susceptibility, can influence the etiology of tendinopathy. The observed phenomenon can generate significant physical and economic difficulties for the population [159]. The secretion of some of the pro-inflammatory mediators, such as TNF-α, COX-2, IL-1β, MMP-1, and IL-6, is of utmost importance in the progression of the underlying disease mechanisms [160]. QC had a protective impact on tendinopathy in rats induced by collagenase. QC inhibits oxidative stress, inflammation, autophagy, matrix degradation, and apoptosis [33]. Similarly, it has been found that QC can hinder oxidative stress along with minimizing tendon adhesion upon cutting and repairing in rats with high levels of GPx and SOD, as well as low levels of MDA. QC exhibits preventive effects against collagenase-induced tendon injury in rats [161]. The overall antioxidative effect of QC on tendons is illustrated in Figure 3.

**Figure 4.**
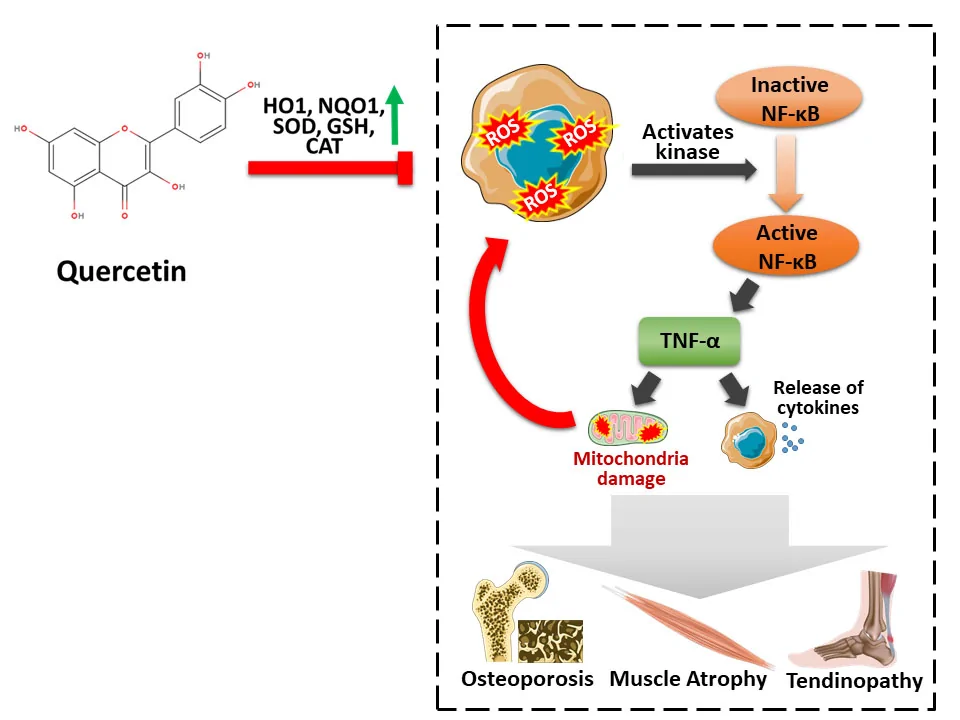
**Mechanism of action by QC for inhibiting ROS**. Increased ROS production activates various kinases and induces the activity of transcription factor NF-κB. TNF-α activated by ROS causes mitochondrial damage, resulting in persistent ROS overproduction. This excess ROS production leads to osteoporosis, muscle atrophy, and tendinopathy. QC has the potential to inhibit ROS production by activating HO-1, NQO1, SOD, GSH, and CAT enzymes which assimilate ROS species in the cells, providing a protective effect.

Taken together, QC can mitigate oxidative stress by releasing enzymes related to the oxidative process and can positively affect bone, muscle, and tendons (Fig. 4). The antioxidant effects of QC on the musculoskeletal system have been summarized in the bone, muscle, and tendons (Table 2).

## 7. Conclusion and future perspectives

The skeletal system is highly dynamic and can regenerate remarkably in response to stress, microdamage, and fractures. During aging, the homeostasis of the skeletal system undergoes physiological alterations, affecting delicate equilibrium between various types of cells of the musculoskeletal system. Conventional treatments have unwanted side effects that restrict their effectiveness. Therefore, safer therapeutic options are warranted for treating aging diseases related to the musculoskeletal system.

Numerous scientific investigations are now being conducted to create musculoskeletal protective compounds that are derived from natural substances. QC, a natural dietary bioflavonoid, has been shown to possess remarkable bioactive abilities that positively affect the musculoskeletal system. The varied qualities of QC, extensively discussed in this comprehensive review, demonstrate its incredible potential in boosting musculoskeletal health, with a particular focus on its robust antioxidant abilities. QC has demonstrated promise in alleviating age-related deleterious effects on bone, muscle, and tendon. It has been noted that it helps maintain bone density, promotes muscle regeneration, and offers protection against oxidative stress and inflammation in tendons. The ability of QC to counteract harmful free radicals and reduce oxidative damage is essential in maintaining the structural integrity of these critical musculoskeletal components. However, understanding the pharmacokinetics of QC is crucial for understanding its effects on the body and its potential as a treatment for enhancing bone health.

The hydrophobic property of QC makes it challenging to deliver orally. The bioavailability of oral QC is relatively low. Moreover, the quantity and quality of dietary fat influence QC absorption in the gastrointestinal tract [164]. Studies have shown that oral bioavailability is below 17% in animals and 2% in humans [165, 166]. However, in natural food sources, such as onions, QC is found as QC-3-glucoside. As determined by the octanol-water partition coefficient, the body more readily absorbs QC-3-glucoside than QC aglycone as it is water-soluble [167, 168]. QC exhibits rapid oral clearance with excretion, owing to its short half-life in the bloodstream. As 90% water-content mucus layer covers QC, it has low gastrointestinal absorption and is metabolized in the liver after entering the body [169, 170]. To overcome the problem of low bioavailability, researchers have recently explored delivery methods like hydrogel, solid dispersion, crystal engineering technology, and nanoformulations utilizing QC [171, 172]. These formulations have shown the capacity to boost the absorption of QC by the epithelial system and improve its distribution to the desired location. Encapsulation of QC using hydrogel systems [173-176] and amorphous solid dispersions with cellulose acetate suberate [177]/ or hydroxypropyl methylcellulose [178] can highly improve the bioavailability of QC. QC-soy phytosome complex solid dispersion increases the antioxidant effect via the Nrf2 signaling pathway [179], and QC-cyclodextrin complexes significantly increase solubility [180]. Co-crystal formation by combining QC with other flavonoids, including baicalein and kaempferol, enhanced solubility and biological activity [181, 182]. A mechanochemical co-amorphous system processed QC-L-arginine, increasing solubility and efficacy [183]. Solid lipid nanoparticles with QC on oral administration to mice (50 mg/ml) showed improved bioavailability through gastrointestinal absorption [184]. Three nanoparticle delivery systems, namely solid lipid nanoparticles, lipid nanoemulsions, and nanostructured lipid carriers, have an encapsulation efficiency of approximately 90% and maximum bioaccessibility [185]. QC-loaded Poly(lactic-co-glycolic acid) nanoparticles show enhanced stability and sustainable release [186]. Flavosomes (flavonoids (such as QC) + lysosomes) were synthesized for the skin delivery system to improve bioavailability through interaction with cell membranes [187]. Studies have found that conjugation of QC with polymeric or metal nanoparticles is the most effective approach for enhancing its uptake [188, 189]. QC-loaded Poly(lactic-co-glycolic acid) nanoparticles show enhanced stability and sustainability. In the future, strategies like chemically combining or enclosing QC within a biopolymer or synthesizing QC with enhanced water-solubility, resistance to degradation in the digestive system, and so on can be employed in drug delivery systems to overcome the limitations of QC and proposed as a potent drug for the treatment of musculoskeletal disorders.

**Table 2:** Antioxidative effects of QC on various physiological processes and diseases.

Diseases/Activities	Antioxidative effect of QC	Reference
**Osteoporosis**	Inhibits RANKL mediated osteoclastogenesis and promotes osteoblastogenesis	[22]
**Rheumatoid arthritis**	Inhibits the expression of the enzyme adenosine deaminase	[19]
**Osteoblast differentiation**	Controls NF-κB and activator protein 1 (AP-1) transcriptional activity.	[162]
**Muscle growth**	Activates the level of Nrf2 and upregulates the expression of several antioxidant enzymes	[163]
**Muscle atropy**	Inhibits the level of p38 MAPK, ERK and NF-κB	[31]
**Achilles tendons**	Decreases the expression of NO	[161]

Randomized controlled trials show that QC supplementation may improve metabolic syndrome components. However, the results are not uniform across all studies. An individual's response may differ, and variables such as dose, supplementation duration, and other health issues may influence the desired outcomes. As far as we know, there have not been many studies that have examined this issue. A recent study reported that the intake of QC may significantly decrease fasting blood glucose and systolic blood pressure without inflicting remarkable changes in other components of metabolic syndrome [190]. Other research has shown that the intake of flavonoids has a beneficial effect on the aging process, specifically in the heart and liver organs, and thus might be beneficial in mitigating long-term susceptibility to cardiovascular or hepatic disorders [191]. These findings suggest that long-term intake of QC for musculoskeletal diseases might be beneficial for improving metabolic syndromes and aging organs. There is currently no precise data on the impact of gender and age on the bioavailability of flavonoids [192]. A noteworthy finding reported that the bioavailability of QC derived from QC-3-rutinoside was higher in women than in men [193]. However, more studies are required to assess the efficacy and safety of QC affected by variable factors like age, gender, and pre-existing diseases in the human population.

Another aspect that needs attention is the dose-dependent response analysis of QC to understand the optimal dosage of QC for maximum efficacy with minimal side effects for clinical applications. A dose-dependent effect of intramuscular injection of QC (10μg,100μg, and 1000μg/kg body weight) was evaluated in the femoral bone microstructure of 5-month-old female rabbits. Morphometric analysis of compact bone showed that a high dose of QC administration significantly reduced the size of primary osteons’ vascular canals in female rabbits. However, the sizes of the secondary osteons significantly decreased in all groups administered with QC, ruling out any dose-dependent QC role. The researchers suggested a reduction in the collagen fibrils in the secondary osteons by QC [194]. A similar dose-dependent effect of intramuscular QC administration was studied in the femur of 5-month-old male rabbits. The study showed a dose-dependent positive effect of QC in qualitative and quantitative histological characteristics of femoral bone. A dose-dependent reduction in the sizes of primary osteons’ vascular canals and secondary osteons was observed in QC-administered male mice. Unlike in female rabbits, a dose-dependent reduction in the sizes of secondary osteons of femur bone was observed in male rabbits. This variation was suggested to be due to the differences in growth and modeling of the femur in males and females, which are controlled by factors like genetic attributes, sex-specific steroids, and many other sources. It can be inferred that administering QC by intramuscular injection has different effects on the microstructure of compact bone in adult rabbits, depending on the sex and dosage. However, histological qualitative bone anatomy in humans shows a limited sex-related variance, and thus, results of QC dose dependency may vary in humans [195]. The dose-dependent effect of QC (500 and 200 mg) on the contractile properties of muscle and motor unit firing patterns in men and women was also compared. Both 500 and 200 mg QC ingestions affected motor unit firing patterns, and QC action was found to be at least partially dosage-dependent [196]. Intriguingly, higher dosages of QC can have undesired influences on osteoblast-specific genes, growth, and mineralization in various in vitro experiments [28, 197-199]. Even the particular kind and placement of the sugar residue attachments to the flavonoid might affect the efficacy of QC [200].

Although there is considerable debate over the safety of QC, the prevailing consensus is that QC is non-toxic. The free QC aglycone is the most common form of isolated QC sold as a dietary supplement, with dosages surpassing the recommended daily allowance of 1000 mg d^-1^ (but usually 500 mg)[201]. Recently, it was observed that even a dose of 2000 mg/day was efficiently tolerated without showing any drug-related adverse events [202]. A study revealed that QC exhibits no acute toxicity, mutagenicity, or chronic or subchronic toxicity in both in vivo and in vitro settings. Conclusions were made from the Ames test, acute toxicity test results, mouse spermatozoa aberration test, mouse bone marrow cell micronucleus test carried out on 30-day feeding in rats, and the 56-day feeding test in laying hens [202]. Most human intervention studies on QC have focused on administering high doses of QC aglycone orally, alone or in combination with high doses of vitamin C, and sometimes with lower quantities of niacin/nicotinamide. These levels are far greater than what is generally received via the food. A few studies have also been conducted where QC has been administrated with other bioactive molecules like curcumin, bromelain, cinnamon bark extract, or green tea extracts [203-205]. Combining QC with other antioxidants like vitamin C raises doubts about whether vitamin C may mitigate the pro-oxidative effects of QC. In human studies, QC did not have pro-oxidative effects at 500-1000 mg d^-1^ doses for 3-12 weeks. However, it remains unclear if high doses of QC may have pro-oxidative effects on the body, especially over time. Human studies have been conducted to assess the toxicity effect of QC with various doses and during a period of intake. A randomized clinical trial showed that daily intake of QC (500 mg/day) for 8 weeks had no adverse effects on patients [206]. Under basal circumstances and in response to high-intensity exercise in humans, the effects of mango leaf extract (Zynamite PX®) along with QC supplementation on skeletal muscle Nrf2 protein levels and Nef2-induced signaling were investigated. Analysis after 48 h of intake of QC observed substantial alterations in resting skeletal muscle signaling, similar to those after exercise training, and partially eliminated stress kinase responses to exercise in trained muscles. No adverse effects on the patients were reported [207]. Based on the World Health Organization's International Agency for Research on Cancer list (last update on December 2023), QC is classified as a Group 3 carcinogen (not classifiable as to its carcinogenicity to humans) despite inadequate human or animal research [208, 209]. However, the animal body has no proven regulatory system for in vitro tests. Moreover, no reliable animal test data exists to support the notion that QC provides no safety risk. Based on existing animal safety assessment experiments within a tolerable dosage range, QC's safety is high.

Natural dietary supplements are considered safe and effective. Natural products and nutritional supplements are routinely used without considering drug-herbal interactions or natural product-drug interactions. Usually, it is expected that natural drug supplement interactions may improve drug effectiveness and reduce toxicity. Nevertheless, unfavorable drug-natural product interactions might also cause adverse side effects. QC has shown the capacity to control the availability of several drugs in both animal and human studies, either via one-time or short-term use [210]. Enhancing the medicine's effectiveness may occur due to a higher drug bioavailability caused by QC. However, this may also raise the probability of experiencing adverse pharmacological effects. Under these circumstances, modifying the administered medication dose may be necessary. Otherwise, the effectiveness of the medicine would be diminished because of the decreased bioavailability of QC. QC has been shown to bind to serum albumin competitively, influencing cytochrome P450, glycoproteins, glucuronidase activity, and organic anion transporting peptides, affecting drug absorption, distribution, metabolism, and clearance in vivo [211]. The probable interaction of QC with efflux transporters like P-glycoprotein (P-gp) and multidrug resistance-associated proteins (MRPs) have been evaluated. It was observed that the inhibition of P-gp by QC led to fatal pharmacokinetic flavonoid-drug interactions, causing the death of pigs co-administration with QC (50 mg/kg) and digoxin [212]. Furthermore, QC was shown to prevent the P-gp inhibitors, taxol, and irinotecan efflux from Caco-2 cells [213, 214]. A preclinical investigation showed that the oral absorption rate of irinotecan was enhanced while the biliary excretion of its hazardous metabolite was reduced after the administration of QC [213]. These findings imply that QC may improve chemotherapeutic efficacy and safety when used with anticancer drugs. In vitro study revealed that QC might modulate the function and expression of MRP2. However, in vivo pharmacokinetics analysis found no effect of such association, suggesting insignificant safety concerns related to this pharmacokinetic interaction [215]. QC and a few of its metabolite conjugates also interact with cytochrome enzymes and drug transporters [210]. Therefore, intake of high doses of QC must be carefully evaluated for its interference with the pharmacokinetics of other drugs. Several animal and human studies have been reviewed and summarized for the mixed outcomes on the drug bioavailability from the QC-drug interaction with a single dose or repeated dosage of 300-1500 mg QC d^-1^ [201, 211]. Nevertheless, the therapeutic importance of altered drug bioavailability by QC remains uncertain. QC has the ability to enhance the absorption of medicine, which might result in improved effectiveness. However, this may also contribute to adverse effects, necessitating modifications to the dosage. Essentially, a substantial quantity of QC does not always ensure its effectiveness. Thus, when determining the optimal dosage and form of QC for its potential effect on human bone health, it is essential to thoroughly assess and confirm its efficacy in clinical trials, and more rigorous studies are required to assess this aspect.

**Table 3:** Clinical trials involving QC for musculoskeletal health (https://clinicaltrials.gov/).

Tissue	Trial No.	Phase	Disease	Remark
**Bone**	NCT05371340	Not Applicable	Postmenopausal osteoporosis	The impact of qc on inflammatory markers and bone health
	NCT06018467	Phase 2	Osteopenia, osteoporosis	Senolytics to improve osteoporosis therapy by using dasatinib and qc
**Muscle**	NCT04258410	Phase 4	Menopause related conditions	Qc for cardio-skeletal muscle health and estrogen deficiency
	NCT05730660	Not Applicable	Chronic fatigue syndrome	Qc phytosome for chronic fatigue syndrome
**Tendon**	No studies reported to date			
**Other parts of the musculoskeletal system**	NCT01720147	Phase 1	Fanconi anemia	A pilot study on qc in children with fanconi anaemia
	NCT03476330	Phase 2	Bone marrow failure	Chemoprevention of squamous cell carcinoma with qc in patients with massive bone marrow failure

Recently, a few studies have been listed in clinical trials for the efficacy of QC in the musculoskeletal system (Table 3). Thus, by harnessing the positive effect of QC on the musculoskeletal system, QC might provide a therapeutic option for the treatment of age-related musculoskeletal disorders such as osteoporosis, sarcopenia, and tendon-related diseases. Findings from in vitro studies only provide an overview of mechanistic aspects, keeping in mind that in vitro studies mostly use high nonphysiological QC concentrations and cannot take into account the complex pattern of QC kinetics (involving processes of metabolism, transport, and distribution) which occur in vivo. For a more complete understanding of QC pharmacological effects and safety, clinical studies and in vivo investigations should be combined. To avoid erroneous results, the QC-interfering effect must be considered while investigating its interaction with other drugs or substances. Thus, more studies are required to examine the pharmacological effects, clinical outcomes, patents, commercial viability of QC products, and strategies for enhancing the bioavailability of QC by using nanoformulation to assert it as a potent drug for human health.
